# Vaccination Coverage with Selected Vaccines and Exemption Rates Among Children in Kindergarten — United States, 2020–21 School Year

**DOI:** 10.15585/mmwr.mm7116a1

**Published:** 2022-04-22

**Authors:** Ranee Seither, Jessica Laury, Agnes Mugerwa-Kasujja, Cynthia L. Knighton, Carla L. Black

**Affiliations:** ^1^Immunization Services Division, National Center for Immunization and Respiratory Diseases, CDC; ^2^Association of Schools & Programs of Public Health, Washington, DC. ^3^Certified Technical Experts, Inc., Montgomery, Alabama.

State and local school vaccination requirements serve to protect students against vaccine-preventable diseases (*1*). This report summarizes data collected for the 2020–21 school year by state and local immunization programs* on vaccination coverage among children in kindergarten in 47 states and the District of Columbia (DC), exemptions for kindergartners in 48 states and DC, and provisional enrollment or grace period status for kindergartners in 28 states. Vaccination coverage^†^ nationally was 93.9% for 2 doses of measles, mumps, and rubella vaccine (MMR); 93.6% for the state-required number of doses of diphtheria, tetanus, and acellular pertussis vaccine (DTaP); and 93.6% for the state-required doses of varicella vaccine. Compared with the 2019–20 school year, vaccination coverage decreased by approximately one percentage point for all vaccines. Although 2.2% of kindergartners had an exemption from at least one vaccine,^§^ an additional 3.9% who did not have a vaccine exemption were not up to date for MMR. The COVID-19 pandemic affected schools’ vaccination requirement and provisional enrollment policies, documentation, and assessment activities. As schools continue to return to in-person learning, enforcement of vaccination policies and follow-up with undervaccinated students are important to improve vaccination coverage.

To meet state and local school entry requirements, parents submit children’s vaccination or exemption documentation to schools, or schools obtain records from state immunization information systems. Federally funded immunization programs work with departments of education, school nurses, and other school personnel to assess vaccination and exemption status of children enrolled in public and private kindergartens and to report unweighted counts, aggregated by school type, to CDC via a web-based questionnaire in the Secure Access Management System, a federal, web-based system that gives authorized personnel secure access to public health applications operated by CDC. CDC uses these counts to produce state-level and national-level estimates of vaccination coverage. During the 2020–21 school year, 47 states and DC reported coverage for all state-required vaccines among public and private school kindergartners;^¶^ 48 states and DC reported exemption data on public school kindergartners and 47 states and DC on private school kindergartners. Overall national and median vaccination coverage for the state-required number of doses of DTaP, MMR, and varicella vaccine are reported. Hepatitis B and poliovirus vaccination coverage, not included in this report, are available at SchoolVaxView (*2*). Twenty-eight states reported the number of kindergartners who were attending school under a grace period (attendance without proof of complete vaccination or exemption during a set interval) or provisional enrollment (school attendance while completing a catch-up vaccination schedule). Thirty states and DC reported the number of kindergartners who had no documentation of any vaccinations or exemptions. Seventeen states reported the number of kindergartners who were out of compliance; these kindergartners did not have complete documentation of having received all required vaccinations but were not eligible for provisional enrollment and did not have documented exemptions for the missing vaccinations. This measure includes those with no documentation at all. All counts were current as of the time of the assessment.** National estimates, medians, and summary measures include only U.S. states and DC. This activity was reviewed by CDC and was conducted consistent with applicable federal law and CDC policy.^††^

Vaccination coverage and exemption estimates were adjusted according to survey type and response rate.^§§^ National estimates measure coverage and exemptions among all kindergartners, whereas medians measure the midpoint of state-level coverage regardless of population size. During the 2020–21 school year, 3,520,205 children in 48 states and DC were reported by immunization programs as enrolled in kindergarten.^¶¶^ Reported estimates are based on 3,187,569 of these kindergartners who were surveyed for vaccination coverage; 3,337,916 for exemptions; 2,467,326 for grace period and provisional enrollment; 1,799,190 for documentation; and 1,049,075 for compliance. Kindergarten enrollment reported by the 48 states and DC was approximately 10% lower than that reported for the 2019–20 school year by 48 states. Potentially achievable coverage with MMR, defined as the sum of the percentage of children who were up to date with 2 doses of MMR and those with no documented vaccination exemption but not up to date, was calculated for each state. Nonexempt students include those who were provisionally enrolled in kindergarten, in a grace period, or otherwise without documentation of complete vaccination. SAS software (version 9.4; SAS Institute) was used for all analyses.

Vaccination assessments varied by state because of differences in required vaccines and doses, vaccines assessed, methods of data collection, and data reported (Supplementary Table 1, https://stacks.cdc.gov/view/cdc/116354). Kindergartners were considered up to date for a given vaccine if they received all doses of that vaccine required for school entry,*** except in nine states^†††^ that reported kindergartners as up to date for any given vaccine only if they received all doses of all vaccines required for school entry. States were asked to report any COVID-19–related impact on kindergarten vaccination measurement and coverage.

Nationally, 2-dose MMR coverage was 93.9% (median = 93.7%; range = 78.9% [DC] to ≥98.9% [Mississippi]). Coverage ≥95% was reported by 16 states and <90% by 7 states and DC (Table). DTaP coverage was 93.6% (range = 78.5% [DC] to ≥98.9% [Mississippi]). Coverage ≥95% was reported by 16 states, and coverage <90% by eight states and DC. Varicella vaccine coverage nationally was 93.6% (range = 78.0% [DC] to ≥98.9% [Mississippi]), with 17 states reporting coverage ≥95% and nine states and DC reporting <90% coverage.

**TABLE:** Estimated* vaccination coverage^†^ for measles, mumps, and rubella vaccine, diphtheria, tetanus, and acellular pertussis vaccine, and varicella vaccine, grace period or provisional enrollment,^§^ and any exemption^¶^^,^** among kindergartners, by immunization program — United States,^††^ 2020–21 school year

Immunization program	Kindergarten population^§§^	% Surveyed^¶¶^	% Vaccine doses			% Grace period or provisional enrollment	% Any exemption	Percentage point change in any exemption from 2019–20 school year	% No documentation^¶¶¶^	% Out of compliance****
			2 of MMR***	5 of DTaP^†††^	2 of varicella^§§§^					
**National estimate^††††^**	**3,520,205**	**90.8**	**93.9**	**93.6**	**93.6**	**2.0**	**2.2**	**−0.3**	**1.0**	**3.4**
**Median^††††^**	**NA**	**NA**	**93.7**	**93.4**	**93.7**	**2.1**	**2.5**	**−0.2**	**0.7**	**2.8**
Alabama^§§§§,¶¶¶¶^	56,974	100.0	≥94.7	≥94.7	≥94.7	NP	1.3	0.1	NR	3.7
Alaska^¶¶¶¶,^*****	9,461	92.5	NR	NR	NR	NR	4.0	−1.9	NR	NR
Arizona^†††††^	76,382	93.4	91.9	92.0	95.5	NR	5.5	0.0	NR	0.6
Arkansas^§§§§§^	37,540	95.6	93.2	92.3	92.8	10.0	2.0	0.1	1.2	NR
California^¶¶¶¶,†††††,§§§§§^	498,214	97.5	95.1	94.7	94.8	0.7	0.5	−0.3	NR	NR
Colorado	63,619	97.3	90.5	90.1	89.4	0.5	≥4.2	−0.7	NR	NR
Connecticut^§§§§,¶¶¶¶^	34,396	100.0	95.3	95.3	95.1	NP	2.6	0.1	NR	NR
Delaware^¶¶¶¶^	10,587	9.2	95.7	94.9	95.3	NR	2.4	NA	0.5	6.1
DC^§§§§,¶¶¶¶^	8,262	100.0	78.9	78.5	78.0	NR	0.3	NA	4.8	NR
Florida^§§§§,¶¶¶¶^	207,026	100.0	≥93.3	≥93.3	≥93.3	3.4	3.1	−0.3	NR	0.2
Georgia^§§§§,¶¶¶¶^	83,191	100.0	≥88.5	≥88.5	≥88.5	0.6	2.9	−0.1	1.0	NR
Hawaii^¶¶¶¶^	13,074	9.3	90.7	91.3	87.2	0.1	3.7	−2.4	0.9	NR
Idaho	22,677	98.3	86.5	86.4	86.2	1.5	8.2	0.6	1.2	7.2
Illinois^¶¶¶¶^	NR	NR	NR	NR	NR	NR	NR	NR	NR	NR
Indiana^¶¶¶¶,^******	78,694	71.4	93.1	83.9	92.8	NR	1.9	−0.3	0.7	16.6
Iowa^§§§§,¶¶¶¶^	39,141	100.0	≥93.4	≥93.4	≥93.4	3.1	2.2	−0.3	NR	1.3
Kansas^¶¶¶¶,§§§§§,¶¶¶¶¶,^******	34,687	32.7	92.6	90.8	91.8	NR	2.0	−0.1	1.3	NR
Kentucky^¶¶¶¶,§§§§§,^******	59,233	86.4	88.9	89.4	88.3	NR	1.0	**−**0.8	5.9	NR
Louisiana^§§§§^	61,912	100.0	96.2	96.9	93.2	NP	1.1	**−**0.4	0.3	NR
Maine	13,477	85.0	94.3	94.0	97.0	NR	4.5	**−**1.4	2.6	NR
Maryland^¶¶¶¶,§§§§§^	65,764	75.6	87.6	89.7	87.3	NR	0.9	**−**0.5	8.3	NR
Massachusetts^§§§§,¶¶¶¶,§§§§§^	60,724	100.0	95.9	95.7	95.4	NP	1.1	−0.2	0.7	5.1
Michigan^§§§§^	106,657	100.0	94.6	95.4	94.2	0.4	3.7	−0.7	0.2	2.8
Minnesota^†††††^	66,007	95.2	89.8	89.3	89.0	NR	≥2.8	−1.0	NR	NR
Mississippi^§§§§,¶¶¶¶,†††††^	34,028	100.0	≥98.9	≥98.9	≥98.9	0.6	0.1	−0.1	0.3	NR
Missouri^§§§§,¶¶¶¶^	63,093	100.0	92.6	92.6	92.1	NR	≥2.5	**−**0.2	1.1	NR
Montana^§§§§,¶¶¶¶^	11,279	100.0	92.9	91.9	91.9	2.0	3.5	**−**0.8	1.1	NR
Nebraska^¶¶¶¶,§§§§§^	25,681	94.8	95.5	96.1	95.1	2.7	2.2	0.0	NR	NR
Nevada^¶¶¶¶^	34,171	94.7	96.1	95.4	95.8	2.1	4.4	0.4	NR	4.1
New Hampshire^¶¶¶¶,^******	10,242	57.0	≥90.8	≥90.8	≥90.8	4.7	2.8	**−**0.3	NR	1.7
New Jersey^§§§§,¶¶¶¶^	100,144	100.0	≥94.3	≥94.3	≥94.3	1.2	2.2	**−**0.4	NR	2.2
New Mexico^§§§§,¶¶¶¶^	20,589	100.0	95.7	95.7	95.3	6.3	0.9	**−**0.6	0.5	NR
New York (including New York City)^ ¶¶¶¶,†††††^	216,804	91.5	98.3	97.8	98.1	1.0	0.1	0.0	0.2	NR
New York City^¶¶¶¶,†††††^	91,920	94.2	97.4	96.6	97.1	0.9	<0.1	0.0	0.4	NR
North Carolina^¶¶¶¶,§§§§§,^******	120,995	89.0	95.2	95.2	95.1	1.7	1.5	**−**0.2	NR	2.6
North Dakota	10,116	99.1	93.3	93.1	93.2	NR	4.2	0.3	0.9	NR
Ohio	128,535	91.1	89.6	89.0	88.7	7.1	2.5	**−**0.3	1.8	NR
Oklahoma^§§§§§^	52,656	90.0	90.5	90.3	96.1	NR	2.4	**−**0.3	1.0	NR
Oregon^§§§§,§§§§§^	39,568	100.0	92.7	91.6	95.1	NR	5.4	**−**1.7	0.6	NR
Pennsylvania	129,307	95.0	95.5	95.9	95.3	3.8	2.7	−0.3	0.1	NR
Rhode Island^¶¶¶¶,§§§§§,^******	10,402	93.0	97.0	96.8	96.7	NR	1.0	−0.3	0.5	NR
South Carolina^¶¶¶¶,¶¶¶¶¶^	56,330	26.5	94.4	95.0	94.2	3.9	2.4	−0.2	0.7	NR
South Dakota^¶¶¶¶^	11,512	99.9	94.6	93.7	94.0	NR	3.4	0.7	NR	NR
Tennessee^§§§§,¶¶¶¶,^******	73,819	100.0	96.6	96.4	96.4	1.0	1.9	**−**0.1	0.5	NR
Texas (including Houston)^ §§§§§,^******	377,840	98.9	95.3	95.0	95.0	1.1	2.3	**−**0.2	0.3	NR
Houston, Texas^§§§§§,^******	39,627	94.9	83.7	83.9	83.1	0.3	1.3	**−**0.2	0.7	NR
Utah^§§§§^	46,247	100.0	91.4	91.1	91.2	4.1	5.1	**−**0.3	0.5	1.7
Vermont^§§§§,¶¶¶¶^	5,535	100.0	94.0	93.6	93.3	5.4	3.2	**−**0.5	NR	NR
Virginia^¶¶¶¶,¶¶¶¶¶^	88,273	2.0	95.8	97.7	94.1	NR	1.5	**−**0.2	0.1	NR
Washington^§§§§,^******	74,931	100.0	94.4	93.2	93.2	0.6	3.3	**−**1.3	NR	5.0
West Virginia^¶¶¶¶,†††††^	NR	NA	NR	NR	NR	NR	NR	NA	NR	NR
Wisconsin^§§§§§,^******	63,486	84.5	≥87.2	≥87.2	≥87.2	5.1	5.2	**−**0.5	0.6	3.1
Wyoming^§§§§,¶¶¶¶^	6,923	100.0	≥90.2	≥90.2	≥90.2	2.4	3.0	**−**0.5	NR	2.1
**Territories and freely associated states**										
American Samoa^¶¶¶¶,††††††^	1,045	100.0	87.7	65.2	56.3	NR	0.0	0.0	NR	NR
Federated States of Micronesia	1,604	96.6	98.4	86.1	Nreq	NR	NR	NA	NR	NR
Guam	NR	NA	NR	NR	NR	NR	NR	NA	NR	NR
Marshall Islands^¶¶¶¶,†††††^	1,016	100.0	99.7	94.4	Nreq	NR	NR	NA	NR	NR
Northern Mariana Islands^§§§§^	830	100.0	94.5	84.2	95.3	NR	0.0	0.0	NR	NR
Palau	NR	NA	NR	NR	NR	NR	NR	NA	NR	NR
Puerto Rico^¶¶¶¶^	26,353	NA	NR	NR	NR	NR	NR	NA	NR	NR
U.S. Virgin Islands	NR	NA	NR	NR	NR	NR	NR	NA	NR	NR

**Abbreviations:** DC = District of Columbia; DTaP = diphtheria, tetanus, and acellular pertussis vaccine; MMR = measles, mumps, and rubella vaccine; NA = not available; NP = no grace period or provisional policy; NR = not reported to CDC; Nreq = not required.

* Estimates adjusted for nonresponse and weighted for sampling where appropriate.

^†^ Estimates based on a completed vaccine series (i.e., not vaccine specific) use the “≥” symbol. Coverage might include history of disease or laboratory evidence of immunity.

^§^ A grace period is a set number of days during which a student can be enrolled and attend school without proof of complete vaccination or exemption. Provisional enrollment allows a student without complete vaccination or exemption to attend school while completing a catch-up vaccination schedule. In states with one or both of these policies, the estimates represent the number of kindergartners who were within a grace period, were provisionally enrolled, or were in a combination of these categories.

^¶^ Some programs did not report the number of children with exemptions, but instead reported the number of exemptions for each vaccine, which could count some children more than once. Lower bounds of the percentage of children with any exemptions were estimated using the individual vaccines with the highest number of exemptions. Estimates based on vaccine-specific exemptions use the “≥” symbol.

** Exemptions, grace period or provisional enrollment, and vaccine coverage status might not be mutually exclusive. Some children enrolled under a grace period or provisional enrollment might be exempt from ≥1 vaccinations, and children with exemptions might be fully vaccinated with ≥1 required vaccines.

^††^ Includes five territories and three freely associated states.

^§§^ The kindergarten population is an approximation provided by each program.

^¶¶^ The number surveyed represents the number surveyed for coverage, except in Alaska. The national number does not include Alaska, which did not report coverage but surveyed 8,756 students for exemptions. In other jurisdictions, exemption estimates are based on 27,421 kindergartners for Kansas, 56,330 for South Carolina, 85,873 for Virginia, and 39,627 for Houston.

*** A majority of states require 2 doses of MMR; Alaska, New Jersey, and Oregon require 2 doses of measles, 1 dose of mumps, and 1 dose of rubella vaccines. Georgia, New York, New York City, North Carolina, and Virginia require 2 doses of measles and mumps vaccines and 1 dose of rubella vaccine. Iowa requires 2 doses of measles vaccine and 2 doses of rubella vaccine.

^†††^ Pertussis vaccination coverage might include some DTP vaccinations if administered in another country. A majority of states require 5 doses of DTaP for school entry or 4 doses if the fourth dose was received on or after the fourth birthday; Maryland requires 4 doses and Nebraska requires 3 doses. The reported coverage estimates represent the percentage of kindergartners with the state-required number of DTaP doses, except for Kentucky, which requires 5 doses of DTaP by age 5 years but reported 4-dose coverage for kindergartners.

^§§§^ A majority of states require 2 doses of varicella vaccine for school entry; Alabama, Arizona, California, Hawaii, Maine, New Jersey, Oklahoma, and Oregon require 1 dose. Reporting of varicella vaccination status for kindergartners with a history of varicella disease varied within and among states; some kindergartners were reported as vaccinated against varicella and others as medically exempt.

^¶¶¶^ Estimates represent the number of kindergartners with no documentation of any vaccinations or exemptions.

**** Students were considered out of compliance if they did not have complete documentation of having received all required vaccinations but were not eligible for provisional enrollment and did not have documented exemptions for the missing vaccinations. This measure included those with no documentation at all.

^††††^ National coverage estimates and medians were calculated using data from 47 states (i.e., do not include Alaska, Illinois, or West Virginia) and DC. National grace period or provisional enrollment estimates and median were calculated using data from the 28 states that have either a grace period or provisional enrollment policy and reported relevant data to CDC. National exemption estimate and median were calculated from data from 48 states (i.e., did not include Illinois or West Virginia) and DC. Other jurisdictions excluded were Houston, New York City, American Samoa, Guam, Marshall Islands, Federated States of Micronesia, Northern Mariana Islands, Palau, Puerto Rico, and the U.S. Virgin Islands. National estimate and median were calculated using data from 30 states and DC for kindergartners with no documentation, and 17 states for kindergartners who were out of compliance. Data reported from 3,187,569 kindergartners were assessed for coverage, 3,337,916 for exemptions, 2,467,326 for grace period or provisional enrollment, 1,799,190 for no documentation, and 1,049,075 for out of compliance. Estimates represent rates for populations of coverage (3,510,744), exemptions (3,520,205), grace period or provisional enrollment (2,608,025), no documentation (2,190,919), and out of compliance (1,109,078).

^§§§§^ The proportion surveyed likely was <100% but is reported as 100% based on incomplete information about the actual current enrollment.

^¶¶¶¶^ Philosophical exemptions were not allowed.

***** Alaska did not report kindergarten vaccination coverage because of problems with data collection. Vaccination coverage among children aged 63–75 months in VacTrAK, Alaska’s Immunization Information System, was 70.2% for MMR, 83.0% for DTaP, and 67.1% for varicella vaccine.

^†††††^ Religious exemptions were not allowed.

^§§§§§^ Counted some or all vaccine doses received regardless of Advisory Committee on Immunization Practices recommended age and time interval; vaccination coverage rates reported might be higher than those for valid doses.

^¶¶¶¶¶^ Vaccination coverage data were collected from a sample of kindergartners; exemption data were collected from a census of kindergartners.

****** Did not include certain types of schools, such as kindergartens in child care facilities, online schools, correctional facilities, or those located on military bases or tribal lands.

^††††††^ Reported exemption data for public schools only.

The percentage of kindergartners with an exemption for ≥1 required vaccines (not limited to MMR, DTaP, and varicella vaccines) was 2.2% in 2020–21 (range = 0.1% [Mississippi and New York] to 8.2% [Idaho]), similar to the 2.5% reported during the 2019–20 school year (Table). Nationally, 0.2% of kindergartners had a medical exemption and 1.9% had a nonmedical exemption (Supplementary Table 2, https://stacks.cdc.gov/view/cdc/116355). The percentage of kindergartners provisionally enrolled in kindergarten or within a grace period among the 28 states reporting these data was 2.0% (range = 0.1% [Hawaii] to 10.0% [Arkansas]) (Table).

Among states that reported data for both 2019–20 and 2020–21, MMR coverage and exemptions for ≥1 vaccines decreased in approximately 75% of states; grace period or provisional enrollment increased in 18 of the 28 states reporting this measure (Figure 1). The proportion of students who were not fully vaccinated and not exempt increased in a majority of states. Among states reporting these measures in 2020–21, the proportion of kindergartners attending school with no documentation of required vaccinations or exemptions ranged from 0.1% (Pennsylvania and Virginia) to 8.3% (Maryland); the proportion out of compliance with school requirements ranged from 0.2% (Florida) to 16.6% (Indiana) (Table). Among the 33 states and DC with MMR coverage <95%, all but two could potentially achieve ≥95% MMR coverage if all nonexempt kindergartners who were within a grace period, provisionally enrolled, or out of compliance received vaccination (Figure 2).

**FIGURE 1:**
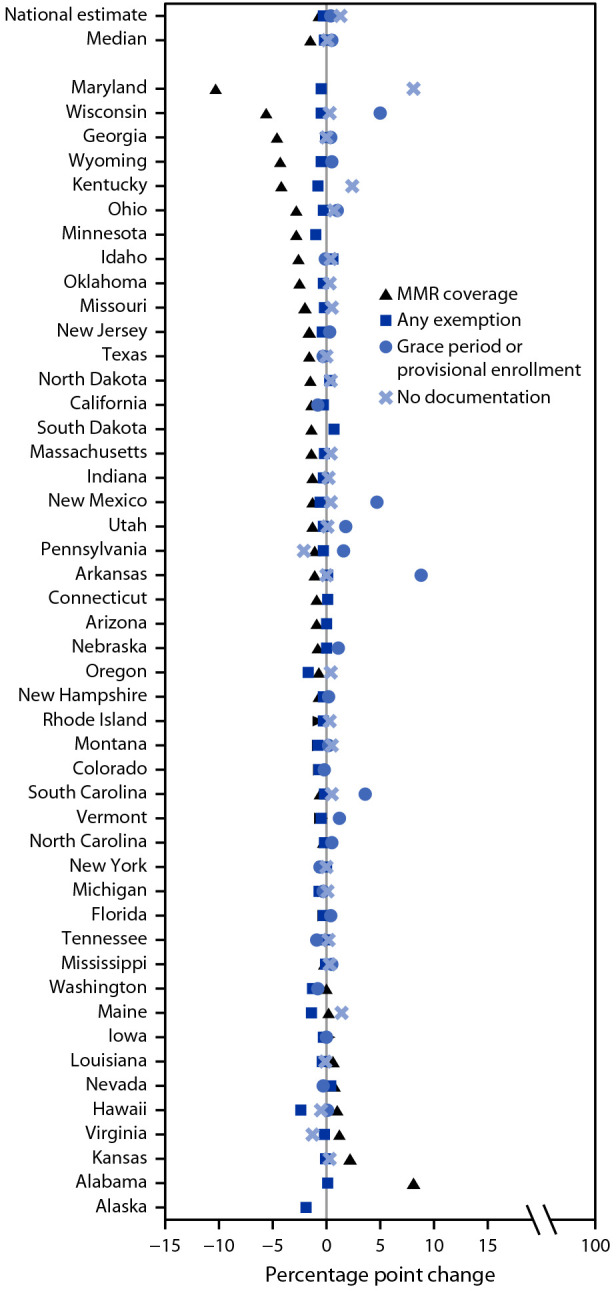
Change in measles, mumps, and rubella vaccine coverage, any exemption, grace period or provisional enrollment, and no documentation among kindergartners* — 47 states,^†^ 2019–20 to 2020–21 school year **Abbreviation:** MMR = measles, mumps, and rubella vaccine. * States are sorted from lowest to highest by change in MMR coverage (n = 46), any exemption (n = 47), grace period or provisional enrollment (n = 28), and no documentation (n = 29). Not all states reported data for all categories. ^†^ Delaware and District of Columbia did not report for any categories for the 2019–20 school year, and Illinois and West Virginia did not report for any categories for the 2020–21 school year. All were excluded from this analysis.

**FIGURE 2:**
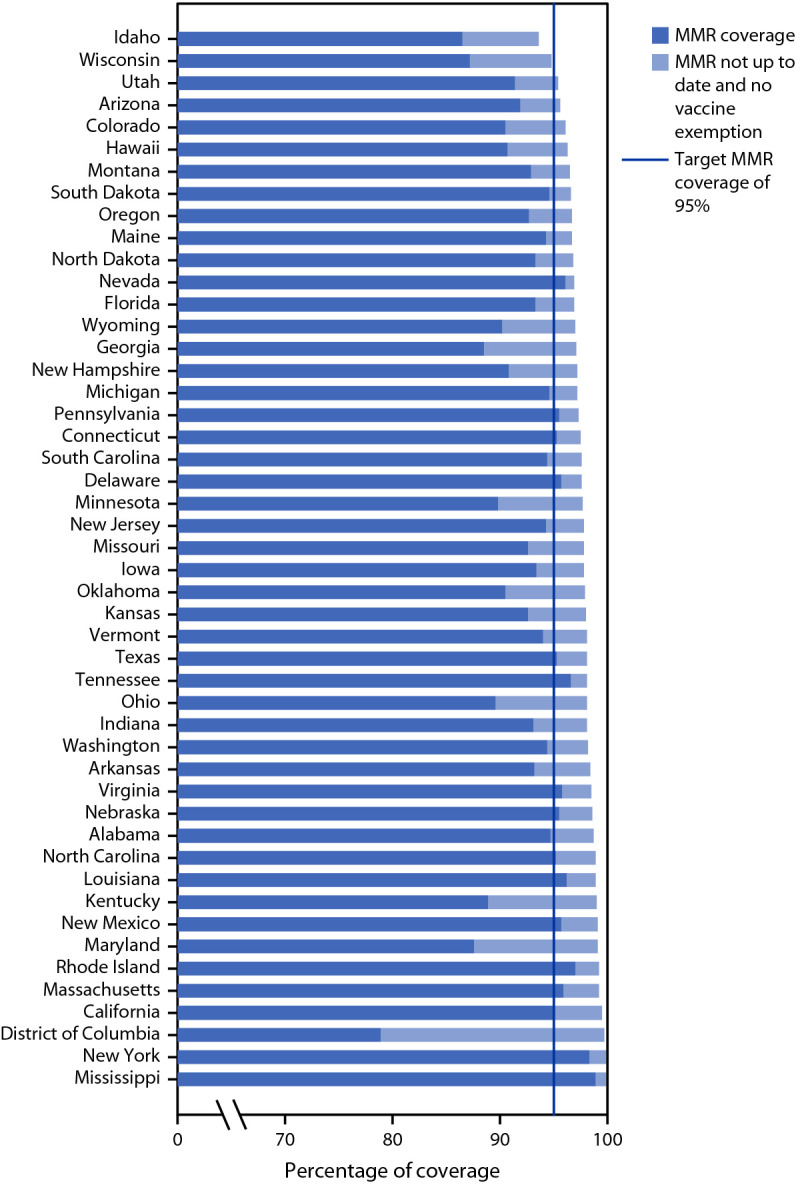
Potentially achievable coverage*^,†^ with measles, mumps, and rubella vaccine among kindergartners, by state — 47^§^ states and District of Columbia, 2020–21 school year **Abbreviation:** MMR = measles, mumps, and rubella vaccine. * States are ranked from lowest to highest by potentially achievable coverage. Potentially achievable coverage was estimated as the sum of the percentage of students with up-to-date MMR and the percentage of students without up-to-date MMR and without a documented vaccine exemption. ^†^ The exemptions used to calculate the potential increase in MMR coverage for Arizona, Arkansas, Colorado, District of Columbia, Idaho, Maine, Massachusetts, Michigan, Minnesota, Missouri, Nebraska, Nevada, New York, North Carolina, North Dakota, Ohio, Oklahoma, Oregon, Rhode Island, Texas, Utah, Vermont, and Wisconsin were the number of children with exemptions specifically for MMR vaccine. For all other states, numbers were based on an exemption to any vaccine. ^§^ Alaska, Illinois, and West Virginia did not report kindergarten vaccination coverage for the 2020–21 school year and are excluded from this analysis.

## Discussion

During the 2020–21 school year, vaccination coverage among kindergartners nationwide was lower than during the 2019–20 school year at approximately 94% (*2*,*3*) for MMR, DTaP, and varicella vaccines, a level just under the target of 95%; coverage for all three vaccines decreased in a majority of states. National MMR coverage among kindergartners fell below the Healthy People 2030 target of 95% (*4*). Reported enrollment and response rates also decreased nationally and in a majority of states (*3*). Some of the decreases in enrollment could be because of schools not reporting these data to state immunization programs, or parents might have decided to have the child delay or skip the kindergarten year. The kindergarten assessment for the 2021–22 school year will include these students if they are enrolled in kindergarten for the 2021–22 school year, but not if they were enrolled in first grade for the 2021–22 school year.

The overall percentage of children with an exemption remained low during the 2020–21 school year at 2.2%; the percentage of children with exemptions decreased in 37 states. Nonexempt undervaccinated students often attend school while in a grace period or are provisionally enrolled; in many states, these policies were expanded either formally or informally during the 2020–21 school year. States described reluctance to schedule and reduced access to well-child appointments, expanded grace period or provisional enrollment, and easing of vaccination requirements for remote learners, reduced submission of documentation by parents, less time for school nurses to follow-up with students missing documentation or vaccines, fewer staff members to conduct kindergarten vaccination coverage assessment and reporting activities, lower response rates from schools, and both extended and compressed kindergarten vaccination coverage data collection schedules, all related to COVID-19 (CDC, School Vaccination Coverage Report, unpublished data, 2021). During the 2020–21 school year, 10% of school principals reported that fewer students were fully vaccinated in that school year.^§§§^ Twenty-seven percent of school nurses reported that fewer students were fully vaccinated in the 2020–21 school year, and 46% of school nurses reported that school vaccination requirements were a somewhat lower or much lower priority compared with previous years (CDC, Impact of the COVID-19 Pandemic on K–12 School Nurses 2020/2021 School Year, unpublished data, 2021). Decreases in vaccine ordering and administration during 2020 also support the measured decreases in coverage (*5*–*8*).

The findings in this report are subject to at least five limitations. First, comparison between states is limited because of variation in states’ requirements such as vaccine required, number of doses required, date required, and type of documentation accepted; data collection methods; exemptions allowed; and definitions of grace period and provisional enrollment. Second, representativeness might be negatively affected because of data collection methods that assess vaccination status at different times or miss some schools or students, such as students who were homeschooled. Third, vaccination coverage, exemption rates, or both might be underestimated or overestimated because of inaccurate or absent documentation or missing schools. Fourth, national coverage estimates for the 2020–21 school year include only 47 of 50 states and DC but use lower-bound estimates for nine states; exemption estimates include 48 states and DC but use lower-bound estimates for four states, and grace period or provisional enrollment estimates include only 28 states. Finally, the COVID-19 pandemic response created various barriers that limited the amount and quality of student vaccination data collected and reported by local health departments. These barriers included schools closing or shifting to virtual learning, many states effectively easing vaccination requirements, and the reassigning of state and local health departments’ staff members to response activities.

Among children aged 4–6 years, vaccination coverage is higher among those in kindergarten than among those not yet in kindergarten (*9*). Although coverage among kindergartners was lower in the 2020–21 school year than in 2019–20 for all reported vaccines, vaccination coverage might be lower among kindergarten-age children whose school entry has been delayed. Vaccination coverage could be improved by increased outreach by schools and immunization programs to first-time students, including kindergartners and first graders, and by follow-up with undervaccinated students. As schools return to in-person learning, high vaccination coverage is necessary to continue protecting students from vaccine-preventable diseases.

**Summary**

**What is already known about this topic?**

State immunization programs conduct annual kindergarten vaccination assessments to monitor school entry vaccination coverage with all state-required vaccines.

**What is added by this report?**

For the 2020–21 school year, coverage was approximately 94% for all required vaccines, approximately one percentage point lower than the previous school year. The exemption rate remained low at 2.2%.

**What are the implications for public health practice?**

Disruptions caused by COVID-19 reduced reported enrollment, school response rates, and documentation for the 2020–21 school year. Schools and immunization programs can increase follow-up with undervaccinated students to reduce the impact of COVID-19–associated disruptions on vaccination coverage to protect students during the return to in-person learning.

## Footnotes

* Federally funded immunization programs are located in 50 states and DC, five cities, and eight U.S territories and freely associated states (territories). Two cities reported data to CDC, which were also included in data submitted by their state. State-level data were used to calculate national estimates and medians. Immunization programs in territories reported vaccination coverage and exemptions; however, these data were not included in national calculations.

† National and median vaccination coverage was determined using estimates for 47 states and DC; Alaska, Illinois, and West Virginia did not report school coverage data because of the impact of COVID-19 on data collection. Data from cities were included with their state data. Data from territories were not included in national and median calculations.

§ National and median exemption rates were determined using estimates for 48 states and DC; Colorado, Minnesota, and Missouri did not collect information on the number of kindergartners with an exemption but instead reported the number of exemptions for each vaccine, which could count some children more than once. For these states, the percentage of kindergartners exempt from the vaccine with the highest number of exemptions (the lower bound of the potential range of exemptions) was included in the national and median exemption rates. Washington was unable to deduplicate students with both religious and philosophical exemptions, so the nonmedical exemption type with the highest number of kindergartners (the lower bound of the potential range of nonmedical exemptions) was included in the national and median exemption rates for nonmedical exemptions. Illinois and West Virginia did not report school vaccine exemption data because of the impact of COVID-19 on data collection. Data from cities were included with their state data. Data from territories were not included in national estimates.

¶ Nine states reported coverage and exemption data for at least some homeschooled kindergartners. Alaska and North Dakota reported some homeschool data separately. California included data for students who attend virtual, partial, or full charter schools with some or all online instruction and students receiving individualized education program services who are medically unable to attend school in public school data. California also included data for homeschools with six or more students in private school data. Montana reported homeschooled students in public school data if the students also attend classes or extracurricular activities at a public school. New Mexico and Pennsylvania included all homeschooled students in public school data. Oregon reported data for students enrolled in exclusively online homeschool programs separately; online students of otherwise traditional public schools were included in public school data. South Carolina and Wisconsin include homeschooled students in their public and private school data if the students also attend classes, extracurricular activities, or have other contact with a school.

** Assessment date varied by state and area. Four states were assessed on the first day of school; 13 states were assessed by December 31; 17 states and DC were assessed by some other date, ranging from 30 days after admission to June 23, 2021; and 16 states were assessed on a rolling basis. Maryland ended data collection early because of COVID-19 response activities.

†† 45 C.F.R. part 46.102(l)(2), 21 C.F.R. part 56; 42 U.S.C. Sect. 241(d); 5 U.S.C. Sect. 552a; 44 U.S.C. Sect. 3501 et seq.

§§ A majority of immunization programs that used census or voluntary response provided CDC with data aggregated at the state or local (city or territory) level. Coverage and exemption data based on a census or voluntary response were adjusted for nonresponse using the inverse of the response rate, stratified by school type (public, private, and homeschool, where available). Programs that used complex sample surveys provided CDC with deidentified data aggregated at the school or county level for weighted analysis. Weights were calculated to account for sample design and adjusted for nonresponse for data collected through complex sample design wherever possible.

¶¶ These totals are the summations of the kindergartners surveyed among programs reporting data for coverage, exemptions, grace periods, and provisional enrollment. Data from cities and territories were not included in these totals.

*** All states required 2 doses of a measles-containing vaccine. Six states (Georgia, New Jersey, New York, North Carolina, Oregon, and Virginia) require only 1 dose of rubella vaccine. New Jersey and Oregon require only 1 dose of mumps vaccine, and mumps vaccine is not required in Iowa. Local DTaP requirements varied. Nebraska required 3 doses of DTaP, two states (Maryland and Wisconsin) required 4 doses, and all other states required 5 doses, unless dose 4 was administered on or after the fourth birthday. The reported coverage estimates represent the percentage of kindergartners with the state-required number of DTaP doses, except for Kentucky, which required 5 doses of DTaP by age 5 years but reported 4-dose coverage for kindergartners. Two states (Maryland and Nebraska) require only 3 doses of polio vaccine, all other states require 4 doses unless the last dose was given on or after the fourth birthday. Six states required 1 dose of varicella vaccine; 44 states and DC required 2 doses.

††† Alabama, Florida, Georgia, Iowa, Mississippi, New Hampshire, New Jersey, Wisconsin, and Wyoming considered kindergartners up to date only if they had received all doses of all vaccines required for school entry.

§§§
https://www.cdcfoundation.org/vaccine-triangulation-report?inline
